# Determining the Minimally Effective Dose of a Clinical Candidate Adeno-Associated Virus Vector in a Mouse Model of Hemophilia A

**DOI:** 10.1089/hum.2021.108

**Published:** 2022-04-19

**Authors:** Jenny A. Greig, Melanie K. Smith, Jayme M.L. Nordin, Tamara Goode, Edward A. Chroscinski, Elizabeth L. Buza, Nicole Schmidt, Lisa M. Kattenhorn, Samuel Wadsworth, James M. Wilson

**Affiliations:** ^1^ Gene Therapy Program, Perelman School of Medicine, Department of Medicine, University of Pennsylvania, Philadelphia, Pennsylvania, USA; ^2^ Bayer Healthcare Pharmaceuticals, Berlin, Germany; ^3^ Ultragenyx Gene Therapy, Cambridge, Massachusetts, USA.

**Keywords:** AAV, hemophilia, gene therapy, factor VIII

## Abstract

Hemophilia A, a bleeding disorder, affects 1:5,000 males and is caused by a deficiency of human blood coagulation factor VIII (hFVIII). Studies in mice and macaques identified AAVhu37.E03.TTR.hFVIIIco-SQ.PA75 as a clinical candidate gene therapy vector to treat hemophilia A. In this study, we sought to determine the minimally effective dose (MED) of this vector in a hemophilia A mouse model. Mice received one of four vector doses (3 × 10^11^–1 × 10^13^ genome copies [GCs]/kg) via intravenous tail vein injection; one cohort received vehicle as a control. Animals were monitored daily after vector/vehicle administration. Blood samples were collected to evaluate hFVIII activity levels and anti-hFVIII antibodies. Animals were sacrificed and necropsied on days 28 and 56; tissues were harvested for histopathological examination and blood was collected for serum chemistry panel analysis. We found no significant differences in liver transaminase levels in mice administered any vector dose compared to those administered vehicle (except for one group administered 3 × 10^11^ GC/kg). Total bilirubin levels were significantly elevated compared to the vehicle group following two vector doses at day 56 (1 × 10^12^ and 1 × 10^13^ GC/kg). We observed no vector-related gross or histological findings. Most microscopic findings were in the vehicle group and considered secondary to blood loss, an expected phenotype of this mouse model. Since we observed no dose-limiting safety markers, we determined that the maximally tolerated dose was greater than or equal to the highest dose tested (1 × 10^13^ GC/kg). Since we detected hFVIII activity in all cohorts administered vector, we conclude that the MED is 3 × 10^11^ GC/kg—the lowest dose evaluated in this study.

## Introduction

Hemophilia A is a common hereditary bleeding disorder affecting 1:5,000 males and is characterized by a deficiency of human blood coagulation factor VIII (hFVIII).^1,2^ Depending on the level of hFVIII deficiency, disease severity can range from mild to severe.^1^ Protein replacement therapy via intravenous (IV) infusion of recombinant hFVIII can effectively maintain a mild-to-moderate phenotype, but the requisite frequency is burdensome to patients.^3^ Adeno-associated virus (AAV) vector-based gene therapy for the treatment of hemophilia A may represent an alternative treatment approach, which only requires a single administration.

The major limitation for an AAV-based gene therapy approach for hemophilia A is the size of the hFVIII coding sequence. The native hFVIII protein is a large, multidomain glycoprotein with complementary DNA (cDNA) that exceeds the packaging capacity for recombinant AAV (>7 kb).^4,5^ We have extensively engineered the promoter and enhancer in the expression cassette^6^ to complement the reduction in size of the hFVIII cDNA performed by others (also referred to as F8).^7^ The protein replacement therapy drug—ReFacto^®^ (Pfizer, New York, NY)—was successfully designed to mimic the smallest active form of hFVIII by replacing the B domain with a 14-amino acid SQ linker.^8^ Subsequent codon optimization of this B-domain-deleted (BDD) hFVIII-SQ (hFVIIIco-SQ) resulted in efficient packaging and increased expression from lentiviral^7,9^ and AAV^6,10^ vectors.

We have extensively evaluated AAVrh10 vectors that are designed to drive liver-specific expression of the codon-optimized BDD hFVIII gene within the size constraints of AAV.^6^ Within a total genome size of <5,250 bp, we generated 42 enhancer/promoter combinations of 3 shortened liver-specific promoters and up to 3 liver-specific enhancer sequences. After evaluating hFVIII activity and immunogenicity of the transgene in a mouse model of hemophilia A (FVIII knockout [KO] mice), we performed an additional capsid-specific immunogenicity evaluation.^6^ Based on our analyses, we selected AAVrh10 and AAVhu37 capsids and the E03.TTR and E12.A1AT enhancer/promoter combinations for further evaluation in nonhuman primates.^11^

Upon systemically administering 1.2 × 10^13^ genome copies (GCs)/kg of AAVhu37.E03.TTR.hFVIIIco-SQ.PA75 (AAVhu37 capsid with transthyretin [TTR] enhancer [E03] and promoter and a codon-optimized version of the hFVIII protein, where the B domain was deleted and replaced by a short 14-amino acid linker [hFVIIIco-SQ]) to cynomolgus macaques, we obtained peak hFVIII activity levels of 23.4% of normal.^11^ While the majority of macaques (18/20) developed anti-hFVIII antibodies within 30 weeks of vector administration, two macaques administered with AAVhu37.E03.TTR.hFVIIIco-SQ.PA75 did not develop anti-hFVIII antibodies during the initial phase of the study (up to 30 weeks postvector administration). These activity levels suggested that our AAVhu37-based gene therapy approach would be sufficient to modify severe hemophilia A phenotypes. The present study describes a pharmacology study with safety measurements for our clinical candidate vector, AAVhu37.E03.TTR.hFVIIIco-SQ.PA75, in FVIII KO mice to determine the minimally effective dose (MED) and to support the initiation of a Phase 1 clinical trial in patients with hemophilia A.

## Materials and Methods

### AAV vector production

AAV vector for the pilot dose-ranging study was produced by the Penn Vector Core at the University of Pennsylvania, as described previously.^12^ In brief, plasmids expressing hFVIIIco-SQ from EnTTR.TTR (E03.TTR) were packaged in the AAVhu37 capsid. Dimension Therapeutics (now Ultragenyx Gene Therapy, Novato, CA) produced the vector for the MED study.

### Mice

We obtained FVIII KO mice (B6;129S-*F8^tm1Kaz^*/J) from The Jackson Laboratory (Bar Harbor, ME). A colony was maintained at the University of Pennsylvania under specific pathogen-free conditions; the mice used for the pilot dose-ranging study were derived from this colony. All animal procedures and protocols were approved by the Institutional Animal Care and Use Committee of the University of Pennsylvania. Each cohort included 10 animals. Before the study, we determined five animals per cohort to be the minimal number to enable statistical analysis of study outcome; we included five additional animals per time point to ensure that enough study animals would be available for meaningful analysis in the occurrence of unexpected deaths, antibody generation to the hFVIII transgene, or other unanticipated events.

### Pilot dose-ranging study

Male FVIII KO mice aged 6–12 weeks received an IV injection of 1.5 × 10^10^, 1.5 × 10^11^, 5 × 10^11^, 1.5 × 10^12^, 5 × 10^12^, or 1.5 × 10^13^ GC/kg of AAVhu37.E03.TTR.hFVIIIco-SQ.PA75 via the tail vein. Vector was diluted in phosphate-buffered saline (PBS). The vehicle control group received an IV injection of 100 μL of PBS. We collected plasma on days 7, 14, and 28 by retro-orbital bleeds into sodium citrate collection tubes. Mice were necropsied on day 28.

### MED study

We obtained mice from Jackson Laboratories and housed five animals per cage in disposable microisolator mouse caging with corn cob bedding. Nestlets were provided for enrichment (Innovive, San Diego, CA). We provided certified irradiated Laboratory Rodent Diet 5002 (LabDiet, St. Louis, MO) *ad libitum*. All interventions were performed during the light cycle. Mice were not fasted before blood collection.

In this study, we used male FVIII KO mice (*n* = 100) aged 8–14 weeks, weighing 18.7–28.8 g in body weight. Before dosing, mice were first allocated to groups. Mice in the same cage belonged to the same group (mixing male mice from different cages into a new cage would cause fighting and possible death as these are hemophilic mice). Each group was randomly assigned to one of the dosing groups using an online program (Research Randomizer, www.randomizer.org/form.htm).

FVIII KO mice received an IV injection of 3 × 10^11^_,_ 1 × 10^12^, 3 × 10^12^, or 1 × 10^13^ GC/kg of AAVhu37.E03.TTR.hFVIIIco-SQ.PA75 via the tail vein. Vector was diluted in 0.01% (w/v) Pluronic F-68, 20 mM Tris, 200 mM NaCl, and 1 mM MgCl_2_ (pH 8.0 ± 0.2). The control group received an IV injection of 100 μL vehicle buffer containing no vector.

Plasma was collected on days 7, 14, 28, and 56 via retro-orbital bleeds into sodium citrate collection tubes. Mice were necropsied on days 28 and 56. As the expressed transgene is human FVIII, most mice would likely have mounted an immune response to the transgene by day 56, neutralizing the activity of any further expressed transgene. Therefore, continuing the study past day 56 was not expected to yield additional pharmacological data.

### Clinical chemistries

We collected blood at the time of necropsy by cardiac puncture in labeled serum gel separator brown-top tubes. After allowing the blood to clot for at least 30 min at room temperature, we centrifuged the samples at 3,500 *g* for 5 min at room temperature. The serum was separated and then shipped to Antech GLP (Morrisville, NC) for analysis of alanine aminotransferase (ALT), aspartate aminotransferase (AST), alkaline phosphatase, gamma glutamyl transferase, total bilirubin, direct bilirubin, and total protein.

### hFVIII activity

We measured hFVIII activity in plasma using the Chromogenix Coatest^®^ SP4 kit, according to the manufacturer's protocol (DiaPharma, West Chester, OH).^6^ In brief, this kit works by combining mouse plasma with unknown hFVIII levels with calcium, phospholipids, and factors IXa and X. The rate of activation of factor X to Xa is dependent on the levels of hFVIII in the plasma sample. A standard curve was generated using known concentrations of BDD-hFVIII-SQ (Xyntha; Wyeth Pharmaceuticals, Inc., Dallas, TX).

### Anti-hFVIII immunoglobulin G

We detected immunoglobulin G (IgG) antibodies against hFVIII in mouse plasma at the time of necropsy via enzyme-linked immunosorbent assay (ELISA) as described previously.^6^ Plasma samples were diluted 1/100 or more and values that were fivefold over background levels (naive mouse samples) were considered positive. The data were reported as anti-hFVIII IgG titers. Negative values are denoted as a titer of 1/50 to enable them to be visualized.

### Histopathology

We harvested tissues at necropsy for comprehensive histopathological examination. These tissues included the injection site, right testis, brain, liver, right kidney, lung, heart, and spleen. Tissues were fixed using 10% neutral buffered formalin, paraffin embedded, sectioned, and stained for histopathology using H&E stain. An experienced, board-certified veterinary pathologist evaluated liver sections in a blinded manner using scoring criteria. Histopathology slides for other tissues were evaluated and peer-reviewed for the highest vector dose group and the vehicle control group. If any findings were reported in the highest dose group, the next lower dose group was evaluated, and so on.

### Vector biodistribution

At the time of necropsy, the liver was collected for biodistribution analysis, frozen on dry ice, and stored at less than or equal to −60°C. We extracted DNA and RNA from liver samples and performed quantitative polymerase chain reactions (qPCRs), as described previously.^6^ Using qPCR targeting a vector-specific sequence, we assayed DNA and RNA samples for vector GC and vector-derived hFVIII transgene levels, respectively. Vector GC assay results were reported as GC per microgram of DNA (GC/μg). We then calculated the vector GC per diploid genome, assuming that one microgram of DNA contains ∼2 × 10^5^ diploid genomes.^13^

### Statistical analyses

We calculated and reported the group average and standard error of the mean for the following: ALT, AST, total bilirubin, total protein, hFVIII activity, vector GCs, and hFVIII RNA transcript levels. We compared groups administered with vector and the vehicle control using a Wilcoxon rank-sum test at each time point for ALT, AST, total protein, total bilirubin, hFVIII activity, vector GCs, and hFVIII RNA transcript levels (nonparametric evaluation as the data appeared to be nonnormally distributed). We compared vector-administered groups to each other using a two-sample Wilcoxon rank-sum test at each time point for hFVIII activity, vector GCs, and hFVIII RNA transcript levels. We used linear mixed-effect modeling to compare the overall change between the vector- and vehicle control-administered groups across all time points. No multiple testing adjustment was performed. A *p*-value of <0.05 was considered significant.

## Results

### Pilot dose-ranging study in FVIII KO mice

**Figure 1.**
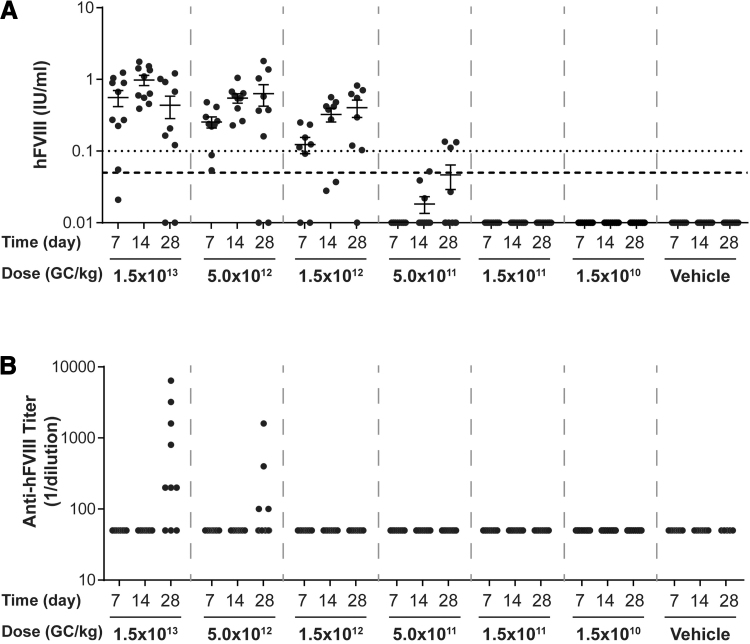
Plasma hFVIII activity levels and anti-hFVIII IgG titers in pilot dose-ranging study. Male FVIII KO mice (*n* = 10/group) were injected IV with 1.5 × 10^13^ GC/kg, 5.0 × 10^12^ GC/kg, 1.5 × 10^12^ GC/kg, 5.0 × 10^11^ GC/kg, 1.5 × 10^11^ GC/kg, or 1.5 × 10^10^ GC/kg of AAVhu37.E03.TTR.hFVIIIco-SQ.PA75 or vehicle control (100 μL of PBS). **(A)** hFVIII activity levels were measured in plasma samples taken throughout the in-life phase of the study and at the time of necropsy by a COATEST assay. *Dashed line* indicates 5% of normal activity; *dotted line* indicates 10% of normal activity. **(B)** Anti-hFVIII IgG titers were measured in plasma samples taken throughout the in-life phase of the study and at the time of necropsy by an anti-hFVIII IgG ELISA. Values that were fivefold over background levels (naive mouse samples) were considered positive. Negative values are denoted as a titer of 1/50 to enable them to be visualized. Graphs show plots of individual mice, with data points and error bars representing mean ± SEM values. AAV, adeno-associated virus; ELISA, enzyme-linked immunosorbent assay; GCs, genome copies; IgG, immunoglobulin G; IV, intravenous; KO, knockout; PBS, phosphate-buffered saline; SEM, standard error of the mean.

Based on the results of our previous studies,^6,11^ we selected the clinical candidate vector, AAVhu37.E03.TTR.hFVIIIco-SQ.PA75. Packaged within the AAVhu37 capsid, this vector had a transthyretin (TTR) enhancer (E03), TTR promoter, and codon-optimized version of the hFVIII protein, in which the B domain was deleted and replaced by a short, 14-amino acid linker (hFVIIIco-SQ). We performed a pilot dose-ranging study in FVIII KO mice to determine the approximate MED (Fig. 1). The FVIII KO mouse is a disease model for hemophilia A. As hemophilia A is an X-linked disease, we used male FVIII KO mice.

We injected IV male FVIII KO mice at 6–12 weeks of age with AAVhu37.E03.TTR.hFVIIIco-SQ.PA75 at doses ranging from 1.5 × 10^10^ GC/kg to 1.5 × 10^13^ GC/kg or with vehicle. We measured hFVIII activity levels and anti-hFVIII IgG titers in plasma samples taken throughout the in-life phase of the study and at the time of necropsy. Following vector administration, we detected a dose-dependent increase in hFVIII activity levels, with no hFVIII activity detected in plasma at doses lower than 5.0 × 10^11^ GC/kg (Fig. 1A). Anti-hFVIII antibodies were only present in mice injected with 5.0 × 10^12^ or 1.5 × 10^13^ GC/kg at day 28 (Fig. 1B). Therefore, the MED of AAVhu37.E03.TTR.hFVIIIco-SQ.PA75 in this research study was 5 × 10^11^ GC/kg.

### MED study rationale and design

Male FVIII mice aged 8–14 weeks received an IV tail vein injection of vehicle control or AAVhu37.E03.TTR.hFVIIIco-SQ.PA75 at one of four doses: 3 × 10^11^, 1 × 10^12^, 3 × 10^12^, or 1 × 10^13^ GC/kg. We performed necropsies on mice 28 and 56 days after administration to capture peak and long-term hFVIII activity.

### Clinical findings

During the study, two mice from the vehicle control group were euthanized for humane reasons (on days 15 and 24). In both cases, we performed a full necropsy and collected tissues for analysis. Histopathology findings pointed to evidence of blood loss in conjunction with the clinical signs, although no direct evidence of hemorrhage or blood loss was observed. During the in-life phase of the study, we recorded clinical observations for 18 out of the 100 mice enrolled in this study that did not affect the outcome. An additional eight mice required supportive care.

### Dose-dependent increase in hFVIII activity

**Figure 2.**
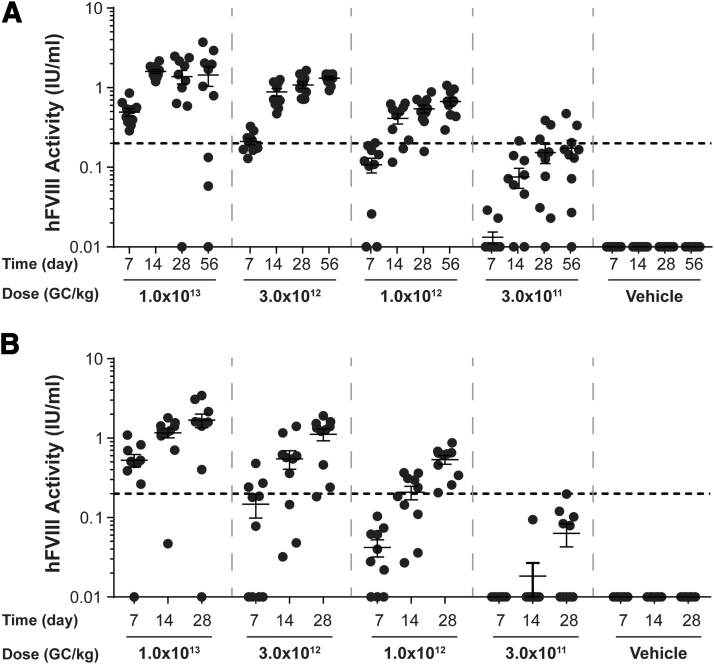
Plasma hFVIII activity levels in vector-administered FVIII KO mice. Male FVIII KO mice (*n* = 10/group) were injected IV with 1 × 10^13^ GC/kg, 3 × 10^12^ GC/kg, 1 × 10^12^ GC/kg, or 3 × 10^11^ GC/kg of AAVhu37.E03.TTR.hFVIIIco-SQ.PA75 or vehicle control. hFVIII activity levels were measured in plasma samples taken throughout the in-life phase of the study and at the time of necropsy by a COATEST assay. Mice were necropsied on day 56 **(A)** or day 28 **(B)**. Graphs show plots of individual mice, with data points and error bars representing mean ± SEM values. *Dashed lines* indicate 20% of normal activity.

**Figure 3.**
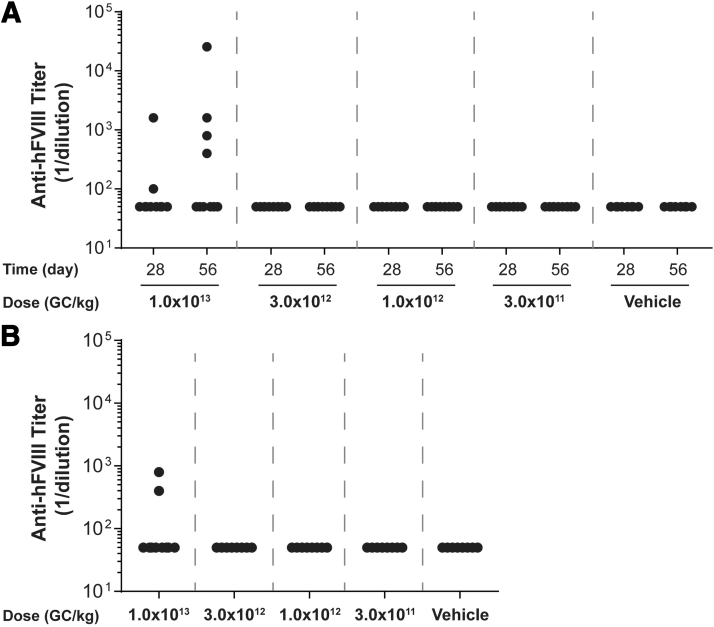
Plasma anti-hFVIII IgG titers in vector-administered FVIII KO mice. Male FVIII KO mice (*n* = 10/group) were injected IV with 1 × 10^13^ GC/kg, 3 × 10^12^ GC/kg, 1 × 10^12^ GC/kg, or 3 × 10^11^ GC/kg of AAVhu37.E03.TTR.hFVIIIco-SQ.PA75 or vehicle control. Anti-hFVIII IgG titers were at the time of necropsy by an anti-hFVIII IgG ELISA. Mice were necropsied on day 56 **(A)** or day 28 **(B)**. Values that were fivefold over background levels (naïve mouse samples) were considered positive. Negative values are denoted as a titer of 1/50 to enable them to be visualized. Graphs show plots of individual mice.

We analyzed plasma hFVIII activity levels throughout the in-life phase of the study. As expected, hFVIII activity levels displayed a dose-dependent increase following IV administration of increasing vector doses (Fig. 2). hFVIII activity increased over the duration of the study from day 7 until the necropsy time point, unless anti-hFVIII IgG antibodies developed (Fig. 3).

For mice necropsied on day 56 postvector administration, the average peak activity level in the high-dose group (1 × 10^13^ GC/kg) was 1.438 IU/mL (equivalent to 143.8% of normal FVIII levels) (Fig. 2A). By day 28 postvector administration, anti-hFVIII IgG antibodies had developed in 2 out of the 10 mice administered the high dose, with an additional two mice in this group developing anti-hFVIII IgG antibodies by day 56 (Fig. 3A). All mice with detectable anti-hFVIII IgG antibodies exhibited a reduction in their individual hFVIII activity levels (Figs. 2 and 3).

For mice necropsied on day 28 postvector administration, the average peak activity level in the high-dose group (1 × 10^13^ GC/kg) was 1.684 IU/mL (Fig. 2B). Similar to mice necropsied at day 56 (Fig. 3A), anti-hFVIII IgG antibodies had developed in two mice in the high-dose group by day 28 postvector administration, which resulted in a decline in their individual hFVIII activity levels (Fig. 3B).

At the lowest dose evaluated in this study (3 × 10^11^ GC/kg), the average peak activity level was 0.173 IU/mL at day 56 (Fig. 2A). As we detected hFVIII activity at all doses of AAVhu37.E03.TTR.hFVIIIco-SQ.PA75 administered in both the day 28 and 56 cohorts, the MED is equal to 3 × 10^11^ GC/kg (the lowest dose administered in this study).

### No correlation between vector dose and elevations in serum ALT or AST levels

**Figure 4.**
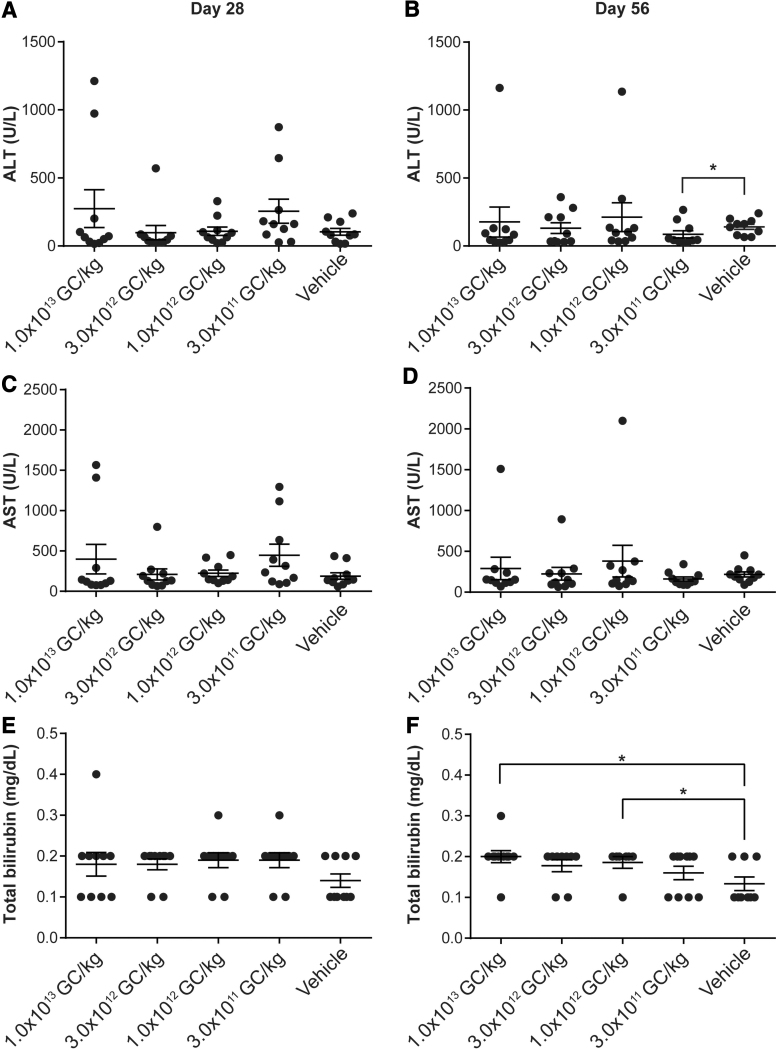
ALT, AST, and total bilirubin levels in vector-administered FVIII KO mice. Male FVIII KO mice (*n* = 10/group) were injected IV with 1 × 10^13^ GC/kg, 3 × 10^12^ GC/kg, 1 × 10^12^ GC/kg, or 3 × 10^11^ GC/kg of AAVhu37.E03.TTR.hFVIIIco-SQ.PA75 or vehicle control. ALT **(A, B)**, AST **(C, D)**, and total bilirubin **(E, F)** levels were measured in serum samples taken at the time of necropsy by Antech GLP. Mice were necropsied on day 28 **(A, C, E)** or day 56 **(B, D, F)**. Values are expressed as mean ± SEM. Groups administered with vector or vehicle control were compared using a Wilcoxon rank-sum test, *p < 0.05. ALT, alanine aminotransferase; AST, aspartate aminotransferase.

Serum chemistry panels were performed on samples collected at necropsy by Antech GLP (Fig. 4). We evaluated the blood chemistry results for statistical differences (*p* < 0.05) in mice administered vector compared to vehicle controls on days 28 and 56 postvector administration. To compare ALT and AST levels in vector- and vehicle control-administered mice, we applied the Wilcoxon rank-sum test. For mice necropsied at day 28, we observed no significant differences in ALT or AST levels in mice administered any vector dose compared to those administered with the vehicle control (Fig. 4A, C). For mice necropsied at day 56, we observed a significant reduction in ALT levels following administration of 3 × 10^11^ GC/kg of the vector compared to the vehicle-treated cohort (Fig. 4B), but observed no significant differences in AST levels (Fig. 4D).

We compared serum total bilirubin levels following administration of vector or vehicle control. Mice necropsied on day 28 displayed no significant differences (Fig. 4E). Mice necropsied at day 56 that were administered 1 × 10^12^ GC/kg or 1 × 10^13^ GC/kg of vector exhibited a significant elevation in total bilirubin levels compared to the vehicle control-administered group (Fig. 4F).

In addition, comparing serum total protein levels revealed significant differences in mice administered with 3 × 10^12^ GC/kg on day 28 (Supplementary Fig. S1A) or 3 × 10^11^ GC/kg of vector at day 56 (Supplementary Fig. S1B).

### Histopathological findings

We harvested tissues from all animals at the time of necropsy, stained them with H&E, and performed a full histopathological analysis. An experienced board-certified veterinary pathologist evaluated the liver sections in a blinded manner using predetermined scoring criteria. Histopathology slides for other tissues were evaluated and peer-reviewed for the highest vector dose group and the vehicle control group. No vector-related microscopic findings were observed (Table 1).

**Table 1.** Summary of histopathology findings for vector-administered FVIII knockout mice

Dose (GC/kg)		1* × *10^13^	3* × *10^12^	1* × *10^12^	3* × *10^11^	N/A	1* × *10^13^	3* × *10^12^	1* × *10^12^	3* × *10^11^	N/A
Necropsy Time Point		Day 28	Day 28	Day 28	Day 28	Day 28	Day 56	Day 56	Day 56	Day 56	Day 56
Liver											
Number examined		10	10	10	10	10	10	10	10	10	10
No abnormalities detected		10	10	10	10	10	10	10	9	10	7
Inflammation, focal	Grade 1	—	—	—	—	—	—	—	1	—	—
EME, focal	Grade 1	—	—	—	—	—	—	—	—	—	1
Hepatocellular necrosis, single cell, multifocal	Grade 1	—	—	—	—	—	—	—	—	—	1
Vacuolation, microvesicular, centrilobular	Grade 2	—	—	—	—	—	—	—	—	—	1
Hepatocellular necrosis, centrilobular	Grade 2	—	—	—	—	—	—	—	—	—	1
Hepatocellular hypertrophy, centrilobular to midzonal	Grade 2	—	—	—	—	—	—	—	—	—	2
Heart											
Number examined		10	0	0	0	10	10	0	0	0	10
No abnormalities detected		8	0	0	0	10	10	0	0	0	9
Mineral, myocardial, focal	Grade 1	2	—	—	—	—	—	—	—	—	—
Mineral, epicardial, focal	Grade 1	—	—	—	—	—	—	—	—	—	1
Fibrosis, epicardial, locally, extensive, focal	Grade 2	—	—	—	—	—	—	—	—	—	1
Pigment-laden macrophages, epicardial	Grade 2	—	—	—	—	—	—	—	—	—	1
Pigment-laden macrophages, perivascular, focal	Grade 1	1	—	—	—	—	—	—	—	—	1
Lung											
Number examined		10	0	0	0	10	10	0	0	0	10
No abnormalities detected		9	0	0	0	7	8	0	0	0	9
Hemorrhage, acute	Grade 1	—	—	—	—	1	—	—	—	—	—
	Grade 2	1	—	—	—	—	—	—	—	—	—
	Grade 4	—	—	—	—	—	1	—	—	—	—
Infiltrate, histiocytic, interstitial, focal	Grade 1	—	—	—	—	1	1	—	—	—	1
Thrombus, acute, focal	Grade 1	—	—	—	—	—	1	—	—	—	—
Foreign body granuloma, focal	Grade 1	—	—	—	—	1	—	—	—	—	—
Spleen											
Number examined		10	0	0	0	10	10	0	0	0	10
No abnormalities detected		10	0	0	0	7	10	0	0	0	8
EME, red pulp	Grade 2	—	—	—	—	2	—	—	—	—	—
	Grade 3	—	—	—	—	—	—	—	—	—	1
	Grade 5	—	—	—	—	1	—	—	—	—	1
Kidney											
Number examined		10	0	0	0	10	10	0	0	0	10
No abnormalities detected		9	0	0	0	9	7	0	0	0	9
Infiltrates, mononuclear cell, interstitial, focal	Grade 1	—	—	—	—	—	2	—	—	—	1
Basophilia, tubule, focal	Grade 1	1	—	—	—	1	3	—	—	—	—
Granular casts, tubular, pigmented, focal	Grade 1	—	—	—	—	1	—	—	—	—	—
	Grade 2	—	—	—	—	—	1	—	—	—	—
Brain											
Number examined		10	0	0	0	10	10	0	0	0	10
No abnormalities detected		10	0	0	0	9	10	0	0	0	8
Squamous cyst, lateral ventricle, forebrain, focal	Grade 1	—	—	—	—	1	—	—	—	—	—

Male FVIII KO mice were injected IV with 1 × 10^13^ GC/kg, 3 × 10^12^ GC/kg, 1 × 10^12^ GC/kg, or 3 × 10^11^ GC/kg of AAVhu37.E03.TTR.hFVIIIco-SQ.PA75 or vehicle control. At necropsy, heart, lung, spleen, kidney, and brain were harvested, fixed using 10% neutral buffered formalin, paraffin embedded, sectioned, and stained for histopathology using H&E stain. Histopathology slides for other tissues were evaluated and peer reviewed for the highest vector dose group and vehicle control group.

AAV, adeno-associated virus; EME, extramedullary erythropoiesis; GCs, genome copies; IV, intravenous; KO, knockout.

Most of the microscopic findings were observed in vehicle-administered animals and were considered potentially secondary to blood loss. However, no macroscopic or microscopic evidence of hemorrhage was observed (Table 1). These findings included centrilobular hepatocellular necrosis (ischemia), extramedullary erythropoiesis in the spleen and liver, epicardial fibrosis with pigment-laden macrophages (consistent with hemosiderin), pigment-laden macrophages with perivascular distribution in the heart, and pigmented (hemoglobin, presumptive) granular casts within renal tubules. Acute alveolar hemorrhage occurred in the lungs of some mice (1/10 mice administered with 1 × 10^13^ GC/kg necropsied at both day 28 and 56, 1/10 mice administered with vehicle control necropsied at day 28). With no histologic evidence of chronicity (*e.g.,* hemosiderophages), we suspected that this finding was perimortem alveolar hemorrhage (potentially secondary to cardiac puncture). The observation of renal interstitial mononuclear cell infiltrates associated with minimal tubule basophilia was considered incidental. Other microscopic findings were incidental, background, or secondary to IV administration, and included myocardial/epicardial mineralization, pulmonary interstitial infiltrates, focal pulmonary foreign body granuloma (hair shaft), focal inflammation in the liver, mononuclear cell infiltrates within the kidney, and a squamous cyst in the brain. The mineralization observed in the heart of a vehicle-administered mouse and two mice from the high-dose group may represent mineralized thrombi, especially given the proximity of the lesions to blood vessels. We found a single acute nonocclusive fibrin thrombus in the lung of one mouse from the high-dose group. The hematopoietic infiltrates in the liver in one vehicle-administered animal were associated with minimal single hepatocellular necrosis.

### Liver vector GC and transgene RNA analysis

**Figure 5.**
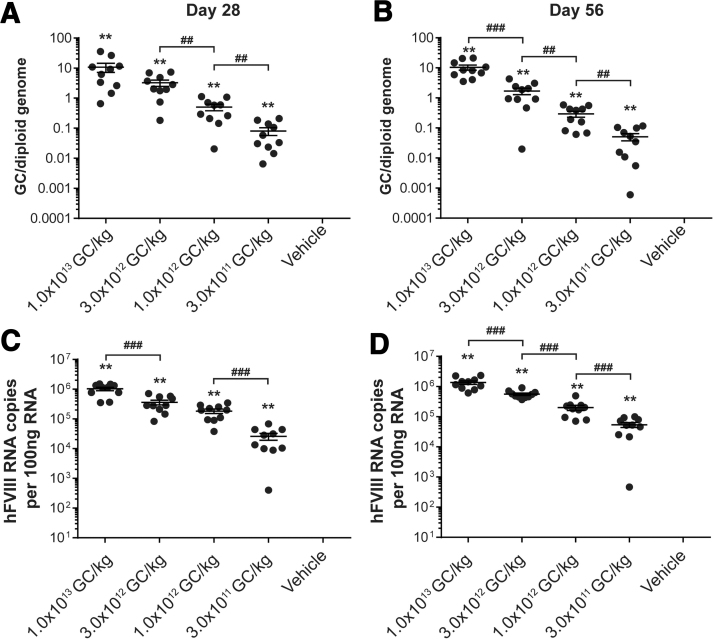
Vector GC and hFVIII RNA transcript levels in livers from vector-administered FVIII KO mice. Male FVIII KO mice (*n* = 10/group) were injected IV with 1 × 10^13^ GC/kg, 3 × 10^12^ GC/kg, 1 × 10^12^ GC/kg, or 3 × 10^11^ GC/kg of AAVhu37.E03.TTR.hFVIIIco-SQ.PA75 or vehicle control. At necropsy, livers were harvested and snap frozen. DNA was extracted for quantification of vector GC. Mice were necropsied on day 28 **(A)** or day 56 **(B)**. Vector GC values are presented per diploid genome. RNA was extracted for quantification of vector transcript levels. Mice were necropsied on day 28 **(C)** or day 56 **(D)**. hFVIII RNA copies are presented per 100 ng of RNA. Values are plotted as mean ± SEM. ns, not significant, **p < 0.01 compared to vehicle; ^##^*p* < 0.01, ^###^*p* < 0.001 compared to next lowest dose.

At the time of necropsy, we collected the liver for biodistribution analysis. We detected a dose-dependent increase in both vector GC and hFVIII RNA levels in the liver (Fig. 5). Using a two-sample Wilcoxon rank-sum test, we compared each vector-administered group for mice necropsied on day 28 or 56 (Fig. 5A, B). We observed significant differences between doses in vector GCs for all vector-administered groups, except for mice administered 1 × 10^13^ GC/kg and 3 × 10^12^ GC/kg and necropsied at day 28 (Fig. 5A).

We performed the same comparisons for hFVIII RNA transcript levels between each vector-administered group (two-sample Wilcoxon rank-sum test, Fig. 5C, D). For mice necropsied on day 28, all vector-administered groups displayed significant differences, except for the comparison between mice administered 3 × 10^12^ GC/kg and 1 × 10^12^ GC/kg (Fig. 5C). For mice necropsied on day 56, all vector-administered groups displayed significant differences in hFVIII RNA transcript levels (Fig. 5D).

## Discussion

In this study, we determined the MED of our clinical candidate vector, AAVhu37.E03.TTR.hFVIIIco-SQ.PA75, in a hemophilia A mouse model. We elected to conduct the experiments in FVIII KO mice (rather than in wild-type C57BL/6 mice) for two reasons. First, using this strain of mice enabled us to evaluate efficacy in parallel with additional safety measurements. Second, we aimed to evaluate potential vector-associated safety signs in the setting of any pathology associated with the defect in FVIII and the associated severe hemophilia and its sequelae. While we did not expect the model to exhibit liver pathology, we were concerned that coagulation deficiencies could influence the response of the host liver to vector.

Following IV administration of AAVhu37.E03.TTR.hFVIIIco-SQ.PA75, hFVIII activity increased over the duration of the study from days 7 to 56, unless anti-hFVIII IgG antibodies developed in individual mice. We detected activity levels of >1.4 IU/mL (equivalent to 140% of normal) at the time of necropsy. It is well established that FVIII levels directly correlate with clinical efficacy.^2^ Indeed, hemophilia A patients are classified into different severity levels depending on the percentage of normal hFVIII; mild (5–40% of normal, 0.05–0.40 IU/mL), moderate (1–5% of normal, 0.01–0.05 IU/mL), and severe (<1%, 0.01 IU/mL). Given this, our results strongly suggest that our gene therapy product would demonstrate clinical efficacy in hemophilia A patients.

While some FVIII KO mice did develop anti-hFVIII IgG antibodies, the relevance of this to the clinical application of this gene therapy approach is unknown as this vector expressed a human protein in mice. Also, we did not determine the region of the hFVIII protein to which the antibodies were binding. Other investigators have used immunodeficient FVIII KO mouse models (either *Rag2*^−/−^ or CD4^−/−^) to evaluate expression and activity in the absence of antibody generation.^14–17^

We found no significant differences in liver transaminases in mice administered any vector dose compared to mice administered vehicle control (except for one group administered 3 × 10^11^ GC/kg). There was a significant elevation in total bilirubin levels following administration of 1 × 10^12^ GC/kg or 1 × 10^13^ GC/kg of vector at day 56 compared to the vehicle-treated group.

Importantly, we did not observe any gross or histological vector-related pathology findings. The majority of the microscopic findings were in mice administered the vehicle control and were considered to point to evidence of blood loss in conjunction with clinical signs, although no direct evidence of hemorrhage or blood loss was observed.

As we observed no dose-limiting vector-related safety measurements, the maximally tolerated dose was greater than or equal to the highest dose tested, which was 1 × 10^13^ GC/kg. Moreover, conducting this study in a hemophilia A animal model allowed us to estimate the MED. We detected hFVIII activity at all doses of the test article administered; hFVIII activity levels were significantly elevated for test article cohorts administered more than 3 × 10^11^ GC/kg at all time points. Therefore, the MED is equal to 3 × 10^11^ GC/kg.

Direct comparison of the MED for AAVhu37.E03.TTR.hFVIIIco-SQ.PA75 to other hemophilia A gene therapy approaches is hampered by a number of factors, including the different mouse models of hemophilia A utilized (immune-competent or -deficient mice). Preclinical evaluations of rAAV8-HLP-codop-hFVIII-V3 in immunocompetent F8^−/−^ mice reported ∼15% of normal hFVIII activity at a dose of 2 × 10^11^ vg/kg,^10^ which is surprisingly similar to that reported here (17.3% of normal hFVIII activity at a dose of 3 × 10^11^ GC/kg). In comparison, evaluation of BMN 270 (AAV5-co-BDD-F8) at a dose of 6 × 10^12^ vg/kg demonstrated only 4.9% of normal hFVIII activity in immunodeficient *Rag2*^−*/*−^ mice and detectable expression in 2 out of 10 double knockout (DKO) mice with mutations in both *FVIII* and *Rag2*.^15^ At a higher dose of BMN 270 (2 × 10^13^ vg/kg), 23.5% of normal hFVIII activity was achieved in DKO mice, which is substantially lower than levels we achieved at a dose of 1 × 10^13^ GC/kg (>140% of normal). Interestingly, in preclinical evaluations of SB-525 (AAV6-co-BDD-F8), levels >330% of normal were seen at a dose of 7.2 × 10^12^ vg/kg in mice that were tolerized to hFVIII (mouse FVIII KO R593C mice contain a hF8-R593C transgene under control of a mouse albumin promoter).^17^

Due to the low MED for this treatment approach for hemophilia A, combined with expression data following systemic administration of the same vector in nonhuman primates,^11^ we believe that this AAVhu37-based gene therapy approach has therapeutic potential in humans. In macaques, the hFVIII activity levels following IV administration of 1.2 × 10^13^ GC/kg would be sufficient to ameliorate a severe hemophilia A phenotype,^11^ thereby reducing or potentially removing the need for recombinant hFVIII infusions. Moreover, a single-administration gene therapy could significantly reduce the cost of treating hemophilia A, particularly in comparison to current IV factor VIII replacement therapy.^18^ Based on this promising preclinical evidence and with an ongoing clinical trial for evaluation (ClinicalTrials.gov Identifier: NCT03588299), we conclude that our clinical candidate vector may represent a long-term, single-administration treatment that has the potential to successfully treat hemophilia A.

## Supplementary Material

Supplemental data
