# Supplementary material for: Determining the Minimally Effective Dose of a Clinical Candidate Adeno-Associated Virus Vector in a Mouse Model of Hemophilia A
Source: Hum Gene Ther. 2022 Apr 19;33(7-8):421–31. doi: 10.1089/hum.2021.108 (PMC9063151; doi:10.1089/hum.2021.108)
Supplement: Supplemental data [file Supp_FigS1.docx]

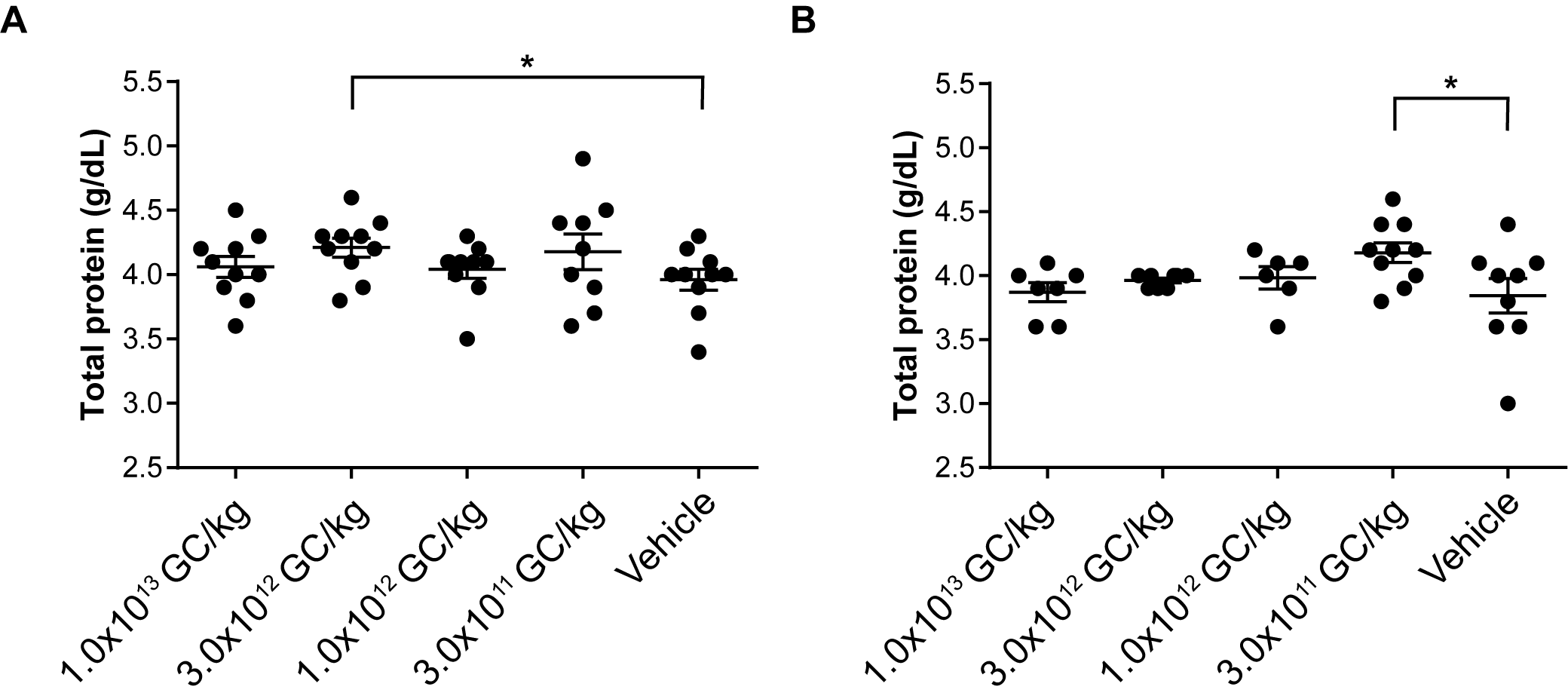


**Supplemental Figure 1. Total protein levels in vector administered FVIII KO mice.**

Male FVIII KO mice (n=10/group) were injected IV with 1x10^13^ GC/kg, 3x10^12^ GC/kg, 1x10^12^ GC/kg, or 3x10^11^ GC/kg of AAVhu37.E03.TTR.hFVIIIco-SQ.PA75 or vehicle control (100 µl PBS). Total protein levels were measured in serum samples taken at the time of necropsy by Antech GLP. Mice were necropsied on day 28 (A) or day 56 (B). Values are expressed as mean ± SEM. Groups administered with vector or vehicle control were compared using a Wilcoxon rank-sum test, **p* < 0.05.
